# Friction Stir-Spot Welding of AA5052-H32 Alloy Sheets: Effects of Dwell Time on Mechanical Properties and Microstructural Evolution

**DOI:** 10.3390/ma16072818

**Published:** 2023-04-01

**Authors:** Mohamed M. Z. Ahmed, Mohamed M. El-Sayed Seleman, Asmaa M. El-Sayed Sobih, Ashraf Bakkar, Ibrahim Albaijan, Kamel Touileb, Ali Abd El-Aty

**Affiliations:** 1 Department of Mechanical Engineering, College of Engineering at Al Kharj, Prince Sattam Bin Abdulaziz University, Al Kharj 11942, Saudi Arabia; i.albaijan@psau.edu.sa (I.A.); k.touileb@psau.edu.sa (K.T.); a.hassibelnaby@psau.edu.sa (A.A.E.-A.); 2 Department of Metallurgical and Materials Engineering, Faculty of Petroleum and Mining Engineering, Suez University, Suez 43512, Egypt; mohamed.elnagar@suezuniv.edu.eg (M.M.E.-S.S.);; 3 Department of Environmental Engineering, College of Engineering at Al-Leith, Um Al-Qura University, Al-Lith 28434, Saudi Arabia; atbakkar@uqu.edu.sa; 4 Mechanical Engineering Department, Faculty of Engineering-Helwan, Helwan University, Cairo 11795, Egypt

**Keywords:** friction stir-spot welding, AA5052-H32 aluminum alloy, dwell time, joint strength, fracture surface, microstructure, electron backscattered diffraction (EBSD)

## Abstract

Friction stir-spot welding (FSSW) as a solid-state joining process for local welding offers a number of benefits for applications in the automotive, aerospace, and marine industries. In these industries, and from an economic point of view, producing spot welds at a low rotating speed and in a short time is critical for saving energy and enhancing productivity. This investigation helped fill a knowledge gap in the literature about FSSW of 4 mm similar lap joints of AA5052-H32 sheet materials, in which welding takes place over a short time period with a slow tool rotation speed. Consequently, the purpose of this work was to investigate the feasibility of FSSW 2 mm thick AA5052-H32 aluminum alloy sheets to produce 4 mm thick similar spot lap joints at various low dwell times of 1, 2, and 3 s and a constant relatively low tool rotation speed of 500 rpm. The introduced heat input for the friction stir-spot welded (FSSWed) lap joints was calculated based on the applied processing parameters. Joint appearance, cross-section macrostructures, and microstructure features of all the spot welds were evaluated. The mechanical properties (hardness contour maps and maximum tensile shear loads) were also examined. The results show that joining 2 mm sheet thickness AA5052-H32 at a low heat input in defect-free similar lap joints could be successfully achieved. The stir zone (SZ) region became wider as the dwell time increased from 1 to 3 s. The hardness value of the SZ was higher than that attained by the AA5052-H32 base material (BM) for all applied dwell times. Especially at 2 s, the hardness of the SZ was approximately 48% higher than that of the BM. This increase in hardness may be attributed to the high grain refinement of the new dynamically recrystallized grain (4 µm) in the SZ compared to the cold-rolled BM grain size (40 µm). Among the tried FSSW process variables, the dwell time of 2 s at a rotation rate of 500 rpm also produced the maximum tensile shear load of 4330 N. Finally, the locations and features of the fracture surfaces of the FSSWed joints were examined using a scanning electron microscope (SEM) and the obtained results were discussed.

## 1. Introduction

The strategy of weight reduction is an essential target of all the transportation industries, mainly to reduce fuel consumption and increase vehicle performance. Consequently, aluminum alloys have received significant attention due to their unique combined properties, especially their lightweight properties [1,2]. Although aluminum alloys possess some advantages, such as high specific strength, high formability, and high resistance to corrosion, joining aluminum alloys has some restrictions [3,4].

Conventional techniques for spot-welding aluminum alloys are mainly riveting and resistance spot welding. There are numerous drawbacks, including reduced efficiency of production and the harsh atmosphere employed in the case of riveting. In addition, high consumption of energy, high current use, and large joint distortion for resistance spot welding are also drawback [2,5]. Mazda Motor Corporation proposed fixed-friction stir-spot welding (FSSW) in 1993 [6,7] dependent on the principle of linear-friction stir welding (FSW), which TWI invented in 1991 [1,3]), and it was successfully used to manufacture the rear door and hood of the sport vehicle Mazda RX-8 [8]. FSSW is considered a green technology due to its high energy efficiency and eco-friendly nature, and because it is a versatile process [7,9]. Furthermore, FSSW is regarded as an excellent replacement for the existing spot-welding processes. Thus, it can be applied in numerous industries, including automobiles, marine, and aerospace applications. The heat required to create proper plastic flow to achieve spot lap joints is mainly generated due to friction between the FSSW tool and the sheets. It is influenced by many variables, such as rotating speed, dwell time, plunge rate, plunge depth, and tool design. The benefits of FSSW over current spot joining methods are that it is a relatively quick operation, does not require filler material, is cost effective, and can weld a range of materials.

Previous works [10,11] showed that the joint properties of FSSWed materials depend initially on the width of the welded area, which is closely related to the tool design and process parameters, especially tool rotation speed and dwell time. Zhang et al. [8] studied the influence of two tool rotational speeds and three different dwell times on the metallurgical and tensile properties of FSSWed AA5052-H112 aluminum alloy sheets of 1 mm thickness using two FSSW techniques. Their results indicated that joint strength is not considerably impacted by dwell time, although it does decrease with increasing rotational speed. In contrast, Uğurlu and Çakan [7] investigated FSSW of AA7075-T6 spot joints fabricated at a constant applied dwell time of 20 s and various tool rotation speeds of 1040, 1320, and 1500 rpm. Their results showed that joint properties improved by increasing the tool rotational rate, and maximum tensile strength was given by the defect-free joint processed at the highest tool rotation speed. In addition, Bozzi et al. [12] remarked two trends in the tensile shear joint strength of FSSWed AA5081-O aluminum strip lap joints processed at tool rotation speeds from 1100–1500 rpm and with a 2.5 s dwell time. Joint strength improved when rotation speed increase from 1100 to 1300 rpm, and a decrease in strength occurred after that with an increase in tool rotation speed, despite the rise in the welded SZ area with an increase in the applied rotation speed. Prasomthong et al. [13] studied the influence of different tool rotating speeds of 2500, 3000, 3500, and 4000 rpm and holding times of 2, 4, 6, and 8 s on the hardness and tensile shear strength of the FSSWed AA5052 Al/C11000 Cu 2 mm thickness dissimilar lap joints. They reported that the weld zone hardness profile of the produced joints showed lower values than that of the BM, and the welding parameters of 3500 rpm rotation speed and 4 s holding time at a 4 mm/min plunge rate showed the highest tensile shear strength of 864 N. Rana et al. [14] studied the influence of the plunge rate in the range of 2 to 12 mm/min on the FSSW of AA5052-H32/HDPE/AA5052-H32 sandwich sheets. The results showed that hook geometry varies significantly with increasing plunge rate, and high joint performance in terms of cross-tension and lap shear load-carrying capacity was attained at plunge rates of 6 and 8 mm/min, respectively. During peel testing, the maximum failure load increased with increasing plunge rate. In addition, they noted an increase in grain size in the SZ, along with an increase in the plunge rate due to an increase in weld peak temperature. Sekhon et al. [15] used experiments designed according to a Taguchi-based L-9 orthogonal array to study the influence of pin geometries (cylindrical, threaded, tapered, and square), tool rotation rates (1000, 1100, and 1200 rpm), and travel speeds (25, 35, and 40 mm/min) on the joint performance of FSW of pure copper and brass butt joints in terms of breaking load and percentage elongation, followed by rotation speed. Square pin geometry at welding conditions of 1200 rpm and 35 mm/min produced a mechanically sound weld joint with the highest joint efficiency. In another work, Tiwan et al. [3] investigated the influence of using two different pin morphologies (cylindrical and step pins) for the FSSW of 3 mm AA5052-H112 alloy in similar lap welds at various tool rotation speeds. They reported that the joint strength prepared using the cylindrical pin was higher than that welded by the step pin with a dwell time of 5 s and rotation speeds of 900 and 1400 rpm. Su et al. [16] reported that two distinguished material flow zones occurred during FSSW. The first was an inner flow zone close to the pin periphery, where the upper welded sheet moved downward in an anticlockwise direction with the rotating pin direction; the second was an outer flow zone, where the lower sheet of the welded materials moved upward and outward in a spiral motion.

In fact, both the FSW and FSSW techniques were achieved by severe plastic deformation [17]. Moreover, the amount of heat input used in the welding process significantly impacted the quality of the produced joints [18]. Furthermore, compared with the FSW technique, FSSW promotes less heat-input energy as a result of the shorter time used (dwell time) [10]. Thus, using considerable heat input is recommended to improve the joint quality of friction stir welds. As a result, aluminum alloy has excellent thermal conductivity, and high heat input may cause the coarse microstructure of the stir zone (SZ), the thermo-mechanically affected zone (TMAZ), and the heat-affected zone (HAZ), which in turn reduced the mechanical strength of the FSSWed joints [6]. Moreover, a question is raised regarding the optimum dwell time to attain enough heat input and achieve well-structured joints.

AA 5052 H32 is a cold-worked aluminum alloy with high weldability, formability, and corrosion resistance in industrial and marine environments [4]. Thus, it is extensively applied in marine structure and automotive applications. There is a need in the marine and automotive industries to friction stir-spot weld 5052-H32 aluminum alloy. Furthermore, the majority of studies have focused on using high rotation speeds (˃900) and high dwell times to achieve FSSW [2,4,19]. From an economic point of view, in terms of saving energy and increasing productivity, it is crucial to produce spot welds at a low rotational speed and over a short time. The current study contributes to the lack of information in the literature related to FSSW of a 4 mm thick similar lap joint of AA5052-H32 under a short range of welding times and a low tool rotation speed. Thus, this work planned to explore the possibility of using FSSW to weld similar 2 mm AA5052-H32 alloy strips in spot joints at a relatively low rotation rate of 400 and at 1, 2, and 3 s of low interval dwell time. To understand the impact of dwell time, as one of the main FSSW process parameters, on the joint efficiency of the produced welds, the joint appearance, cross-section macrostructure, and mechanical properties in terms of tensile shear load and hardness contour map were evaluated, and the main results were discussed. Finally, in order to fully understand how optimized dwell time affects the microstructure features of the AA5052-H32 BM and the SZ of the FSSWed specimens, which achieve the highest tensile shear load-carrying capacity, an electron backscatter diffraction (EBSD) investigation was conducted and discussed.

## 2. Experimental Procedures

### 2.1. Starting Materials

The starting material was an AA5052-H32 alloy sheet that was 1000 mm in length, 1000 mm in width, and 2 mm in thickness. The chemistry and properties of the as-received AA5052 alloy sheet are listed in Table 1 and Table 2, respectively.

### 2.2. FSSW Process Parameters

**Figure 1:**
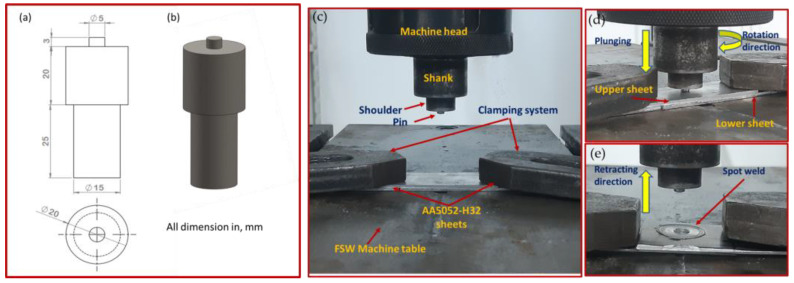
(**a**) Tool drawing (all dimensions were in mm); (**b**) isometric tool image; (**c**)FSSW setup; (**d**) tool rotating and plunging; and (**e**) joint formation and tool retracting.

The FSSW process in the current work used an FSW machine (EG-FSW-M1) for the joining process [20,21]. For spot-welding experiments, the as-received AA5052-H32 sheets were cut into strips that were 10 cm in length, 3 cm in width, and 0.2 cm in thickness. Then, the strips were FSSWed at 1, 2, and 3 s dwell times with a constant tool rotation rate of 500 rpm in similar spot lap joints with a 30 mm overlap. The plunge depth, tilt angle, and plunge rate were all maintained at constant values of 3.2 mm, 0°, and 0.1 mm/s, respectively. The FSSW tool was designed with a flat shoulder of 20 mm in diameter and a cylindrical pin of 5 and 3.2 mm in diameter and length, respectively, as displayed in Figure 1. The welding tool material was manufactured from H13 hot-worked steel. The tool design and material used in this investigation were mainly based on the previous studies conducted by [22,23,24].

**Figure 2:**
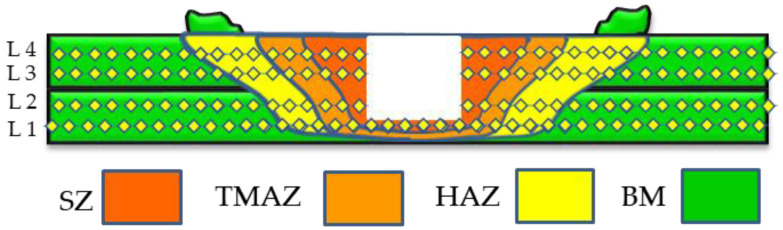
Schematic drawing for the measurement locations during the hardness test of the spot-welded joints.

The FSSWed joints were cut into cross-sectioned samples for macrostructure, microstructure examination, and hardness testing. The sectioned specimens were ground and polished using 0.05 µm alumina suspension. The polished surfaces were etched using Keller’s etcher of 6 mL nitric acid (HNO_3_), 3 mL hydrofluoric acid (HF), and 95 mL distilled water (DW). The optical microstructure was evaluated using an Olympus microscope (Model BX41M-LED, Tokyo, Japan). In addition, the microstructures of the starting material, as well as the FSSWed joint, were investigated using EBSD in a Quanta FEG 250 SEM equipped with a Hikari EDAX-EBSD advanced camera. For the EBSD study, the mechanically polished specimens were electropolished in an electrolyte solution composed of 70 vol.% CH_3_OH and 30 vol.% HNO_3_ at a temperature of −18 °C and 14 V for 55 s. The electrolyte solution used contained 70 vol.% CH_3_OH and 30 vol.% HNO_3_. To represent the hardness values of the FSSWed zones and the BM, the hardness test was achieved on the cross-sections of the FSSWed lap joints to obtain hardness contour maps at the different welding conditions. The hardness measurements were performed via a Vickers hardness testing instrument (Type HWDV-75, made in Osaka, Japan) using the loading conditions of 5 N with a 15 s duration time. The cross-section of the AA5052-H32 FSSWed joint is divided into four lines to present the two welded strips, as shown in Figure 2. A fixed and sufficient distance of 0.75 mm was left between each of the two indentations to avoid the stress field zone.

**Figure 3:**
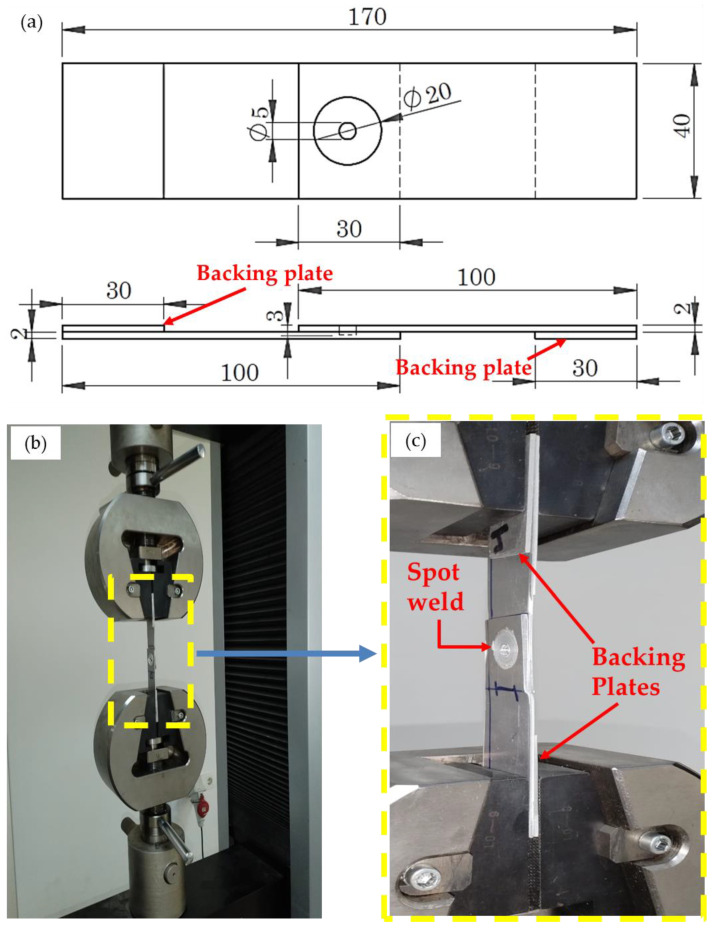
(**a**) Schematic of the spot-welded tensile shear test specimen; (**b**) picture of the specimen during the tensile shear test; and (**c**) high-magnification picture.

To evaluate each joint strength, a universal testing machine of 300 kN capacity (model: WDW-300D, made in Dongguan, China) was used to conduct the tensile shear test. The FSSWed lap joints were tensile tested at a 0.05 mm/s loading rate. The tensile shear test sample of the FSSWed AA5052-H32 alloy lap joint is schematically depicted in Figure 3. The tensile shear test was conducted according to the standard ANS1/AWS/SAE/D8.9-97. During the test, two backing plates of AA5052-H32 were used to ensure the applied axial load. The fractured surfaces of the failed FSSWed lap joints were examined and analyzed.

## 3. Results and Discussions

### 3.1. Surface Morphology of FSSWed Joints

**Figure 4:**
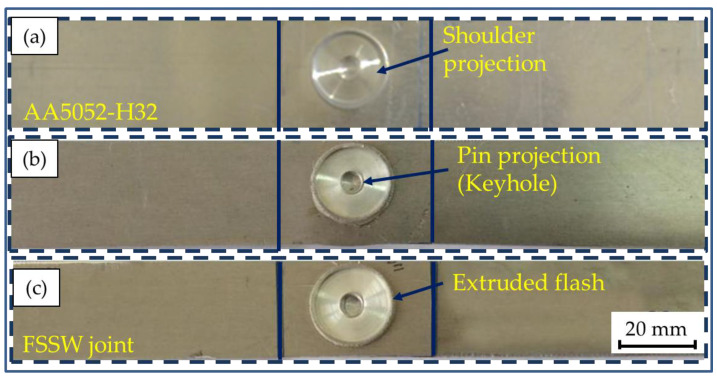
The visual appearance of the AA5052-H32 FSSWed lap joints processed at 500 rpm rotation rate and various dwell times of (**a**) 1, (**b**) 2, and (**c**) 3 s.

The top surfaces of the FSSWed AA5052-H32 similar lap joints processed at 1, 2, and 3 s dwell times with a constant tool rotation speed of 500 rpm are given in Figure 4. It can be said that the suggested spot-welding conditions for processing AA5052-H32 in 4 mm thick lap joints are appropriate without any joint distortion. The FSSWed joints’ distinguishing features of circular indentations of shoulder projection and the formed keyhole are shown for all welding parameters. All the shoulder projections, keyholes, and extruded flash are nearly identical.

### 3.2. Heat Input for Joining Process

The heat input generated during FSSW of AA5052-H32 depends on the tool material and design, feeding rate, pin and surrounding material interface friction coefficient, rotation rate, applied dwelling time, and shoulder plunge depth [25]

The total heat input ($Q_{t}$) for AA5052-H32 FSSW can be obtained using the following equations [25,26]:

(1) $$Q_{t}=K\times \mu \times \frac{2\pi n}{60}\times \frac{F}{K_{A}}\times r_{p}\times t$$

where K is a constant and equals 1.083; μ equals 0.4 (friction coefficient at the pin/surrounding strip material interface) [NO_PRINTED_FORM]; F is the vertical force (N); $K_{A}$ is the applied tool’s shoulder contact area to the total shoulder cross-sectional area during the FSSW procedure; $r_{p}$ is pin radius (m);  n is the tool rotation rate (rpm); and *t* is the used dwell time (s).

(2) $$K_{A}=\frac{\left(\mathrm{tool}\;\mathrm{shoulder}\;\mathrm{radius}\right){\;}^{2}-{\left(\mathrm{pin}\;\mathrm{radius}\right)}^{2}}{{\left(\mathrm{tool}\;\mathrm{shoulder}\;\mathrm{radius}\right)}^{2}}$$

where the given tool shoulder and pin radiuses were 10 and 2.5 mm, respectively.

From Equations (1) and (2):

(3) $$Q=1.1859\times 10^{\left(-3\right)}\times F\times t\times n\;(J)$$

**Figure 5:**
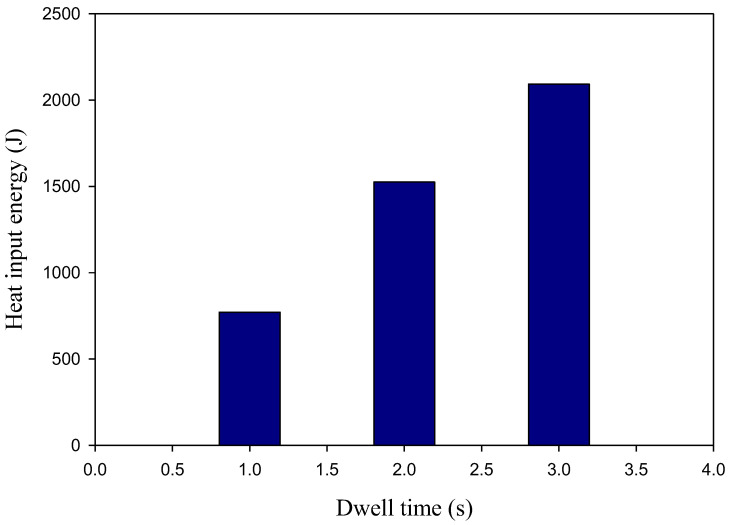
The relation of heat-input energy versus the used dwell time for the AA5052-H32 FSSWed materials processed at 500 rpm.

The $(Q_{t}$) values of the AA5052-H32 FSSW lap welds were plotted versus the dwell time value, as depicted in Figure 5. It can be observed that increasing the dwell time from 1 to 3 s caused a rise in the introduced thermal heat energy value from 771 to 2092 J during the welding procedure.

### 3.3. Macrographs of the FSSWed AA5052-H32

**Figure 6:**
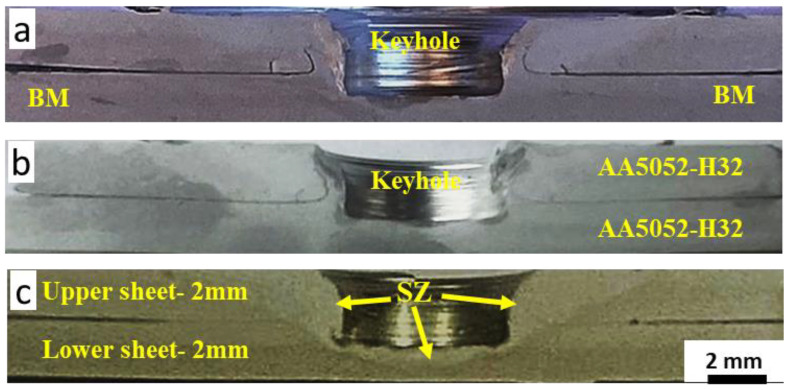
Macrographs of the transverse cross-sections of the FSSWed AA5052-H32 specimens processed at a constant tool rotational speed of 500 rpm and different dwell times of (**a**) 1 s, (**b**) 2 s, and (**c**) 3 s.

Figure 6 illustrates the optical macrographs of the FSSWed AA5052-H32 lap joints cross-sections. The applied spot-welding parameters produce defect-free welds, and the SZ area increased with the increase in dwelling time from 1 to 3 s. The weld zone between the AA5052 aluminum strips was achieved as a result of plastic deformation and material flow caused by pin rotation at applied dwell times of 1 s (a), 2 s (b), and 3 s (c) using a 500 rpm tool rotation rate, as Figure 6 shows. Furthermore, no excessive extruded flash material was observed because of the excellent selection of low heat-input energies based on many experimental trials and published works [27]. The processed FSSWed joints had a distinctive keyhole at the center of the weld; the pin left this hole after the welding process was completed. This keyhole exactly matched the pin geometry and dimensions. According to previous works [3,25,26], three distinct regions develop in the FSSWed area, namely the SZ, the thermo-mechanically affected zone (TMAZ), and the heat-affected zone (HAZ). These three regions are characteristic of the cross-sectional joints processed with friction stir processing (FSP) [28,29], FSW [30], and FSSW [3,8,12].

### 3.4. Hardness Evaluation

**Figure 7:**
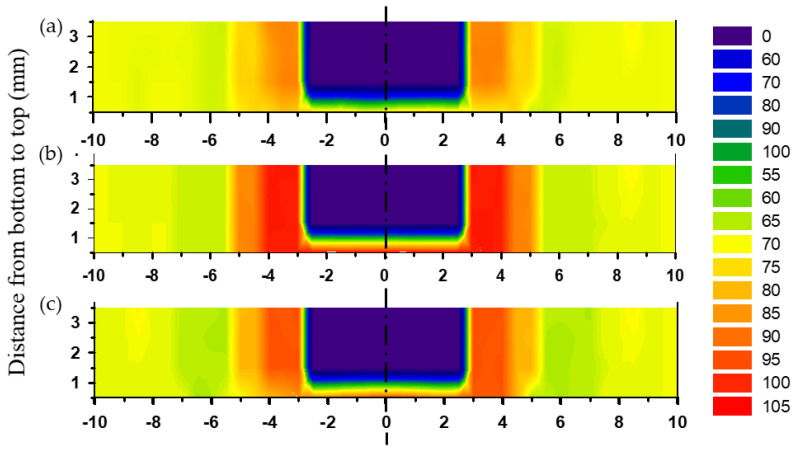
Hardness-colored contour maps of the FSSWed AA5052-H32 joints produced at 500 rpm and various dwell times of (**a**) 1 s, (**b**) 2 s, and (**c**) 3 s.

**Figure 8:**
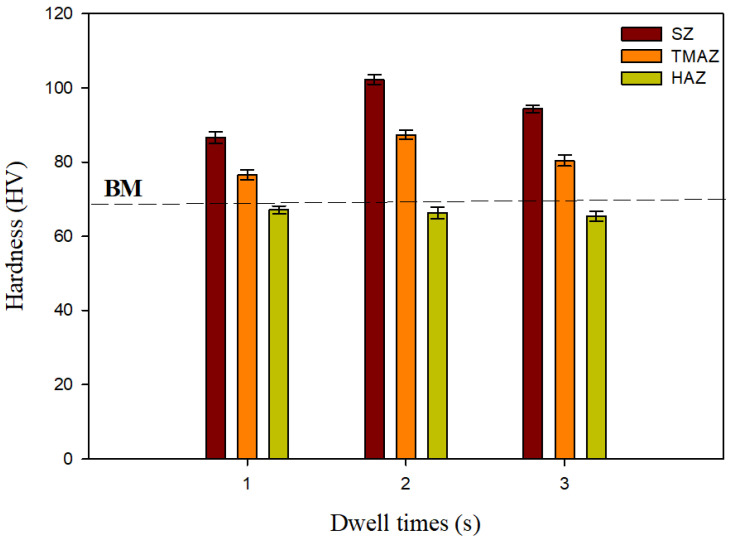
Average hardness in the weld zones (HAZ, TMAZ, and SZ) of the FSSWed AA5052-H32 lap joints achieved at various dwell times (1–3 s) and 500 rpm tool rotation speed.

Hardness was assessed along the cross-sections of the FSSWed AA5052-H32 lap joints, and the results are shown as a contour map with colors denoting different hardness levels. Figure 7 displays the hardness contour maps of the FSSWed joints at a fixed tool rotation rate of 500 rpm using various dwell times. Symmetrical hardness distribution to the centerline of the FSSW keyhole was achieved with the cylindrical pin used at all applied dwell times, 1, 2, and 3 s, as given in Figure 7a–c, respectively. In addition, the hardness values of the TMAZ and SZ of the FSSWed joints at all employed dwell times were significantly enhanced compared to the AA5052-H32 BM. However, the HAZ hardness values were close to or slightly less than that of the BM, depending on the duration of the thermal exposure time, as shown in Figure 7 and Figure 8. These findings are consistent with those reported by previous researchers [3,12,31]. Bozzi et al. [12] found that the SZ of the FSSW AA5182 joints had a higher hardness than the AA5182 BM. The difference in hardness between the SZ and BM was attributed to the finer grain in the SZ than was presented in the AA5182 BM. Using the same approach, Tiwan et al. [3] recorded an increase in the hardness in the SZ compared to both the HAZ and the TMAZ of the FSSWed AA5052-H112 aluminum alloy. They concluded that this drop in hardness of both HAZ and TMAZ was most likely caused by grain coarsening throughout the weld heat cycle; meanwhile, they related the hardness increase in the SZ to the grain refining that occurred due to the dynamic recrystallization process. This dynamic recrystallization process occurred when the aluminum alloys are deformed at high temperatures [32,33]. Kwon et al. [34] also attributed the increase in SZ hardness of the FSSWed A5052-O aluminum alloy over the BM to grain refinement. A finer grain in the SZ was once more linked to the SZ’s better hardness in comparison to the BM. According to the Hall–Petch equation [35], the alloy materials of smaller grain sizes offered higher hardness and yield strength.

In the current work and based on the above discussion, for each FSSWAA5052-H32 joint, the hardness values were found to be at their lowest in the HAZ (Figure 8). This may be attributed to the features of grain structure affected by the applied thermal cycle and strain-hardening release. Meanwhile, the highest hardness value found in the SZ was mostly related to the equiaxed fine grain structure and the intermetallic fragmentation that may occur during stirring. The TMAZ displays lower hardness measurements than that of the SZ but has higher hardness values than HAZ. The increased hardness of the TMAZ above that given by the HAZ is mainly due to the severe plastic deformation experienced. The FSSWed joints processed at 2 s showed the highest hardness values of 102.3 ± 3 in the SZ compared to the BM (69 ± 2) and the other joints processed at 1 s (86.6 ± 1.5) and 3 s (94.3 ± 2.5), as illustrated in Figure 8. This explains that the amount of heat input generated in the SZ at 2 s and 500 rpm (1525 J) is the optimal value to obtain and maintain dynamically recrystallized fine grain structure, and any further increase in heat input leads to a decrease in hardness, as in the case of the welded joint at 3 s, as a result of the expected growth of grains after the completion of the recrystallization process, which may also result in a drop in joint strength.

### 3.5. Tensile Shear Results and Fracture Surfaces

**Figure 9:**
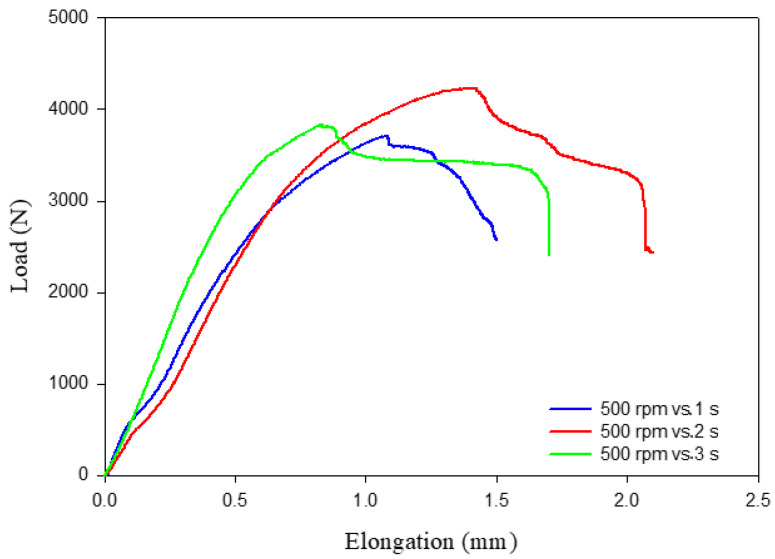
Tensile shear curves of the AA5052-H32 welded joints achieved at 500 rpm rotation speed and dwell times of 1, 2, and 3 s.

**Figure 10:**
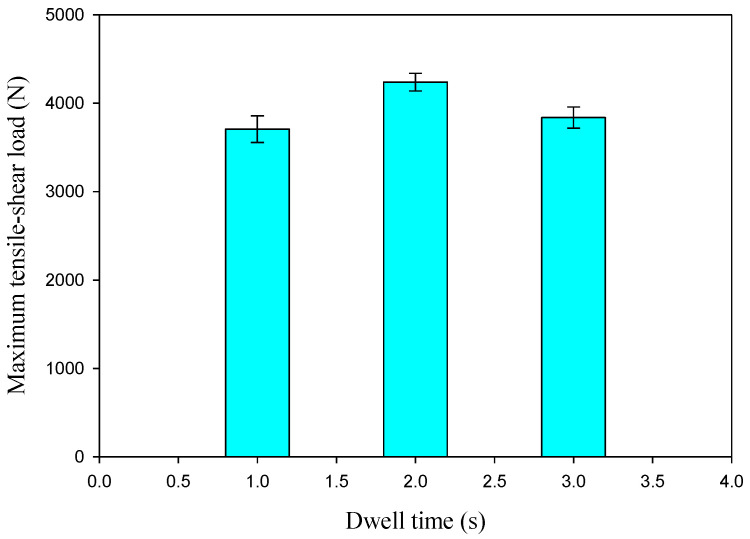
Maximum tensile shear load of the FSSWed AA5052-H32 against the applied dwell times.

When creating new models of automobiles and ships, wagon designers give more consideration to the tensile shear results of the spot-welded connections. As a result, the tensile shear test was performed for all AA5052-H32 joints produced by FSSWed. In many works [26,36,37], it was concluded that the FSSW process parameters govern joint strength. The tensile shear test results of the produced joints at 500 rpm and different dwell times of 1, 2, and 3 s are given in the load-elongation plots in Figure 9. The tensile shear load of the FSSWed joints of AA50520-H32 as a function of the applied dwell time is depicted in Figure 10. Figure 9 and Figure 10 show that the FSSW conditions of a 2 s dwell time and a 500 rpm rotation rate have the maximum tensile shear load of 4330 N compared to the tensile shear loads of the spot-welded joints processed at 1 s (3660 N) and 3 s (3820 N). This increase in joint performance may be ascribed to the lower hook height and the larger fully bonded zone [31,38,39]. In addition, the hardness enhancement in the SZ of the joint spot welded at a 500 rpm rotation speed and 2 s spot dwell time is higher than that measured in the stir zone of other FSSWed materials. The lowest dwell time of 1 s produced insufficient heat input (Figure 5), resulting in inadequate mixing between the two AA5052-H32 strips during the FSSW process. This leads to a decrease in the joint bonded area, making it more likely to separate under loading compared to the other joints processed at 2 and 3 s. Moreover, at the highest dwell time of 3 s (the highest heat-input energy), sufficient mixing is achieved between the two welded sheets, and the extra heat may cause softening (drop in hardness) in the bonded area and increase the HAZ area, which leads to lower joint strength than that processed at 2 s (Figure 10). Accordingly, it can be concluded that the amount of heat input generated as a result of applying welding conditions of 500 rotational speed and 2 s of holding time is sufficient to make a good mix between the two AA5052-H32 sheets and build a welding joint that has the highest hardness and the highest load-carrying capacity.

Based on the results of two different applied tension tests (cross-tension test and tensile shear test), Zhang et al. [40] and Tiwan et al. [3] identified various fracture mechanisms for similar FSSW AA5052-H112 lap joints with different sheet thickness and at different processing parameters. They found that under cross-tension loading, SZ debonding and pull-out take place, but under tensile shear loadings, shear fracture and tensile shear mixed fracture are the fracture mechanisms. They also came to the conclusion that the type of fracture mode is primarily determined by joint performance.

**Figure 11:**
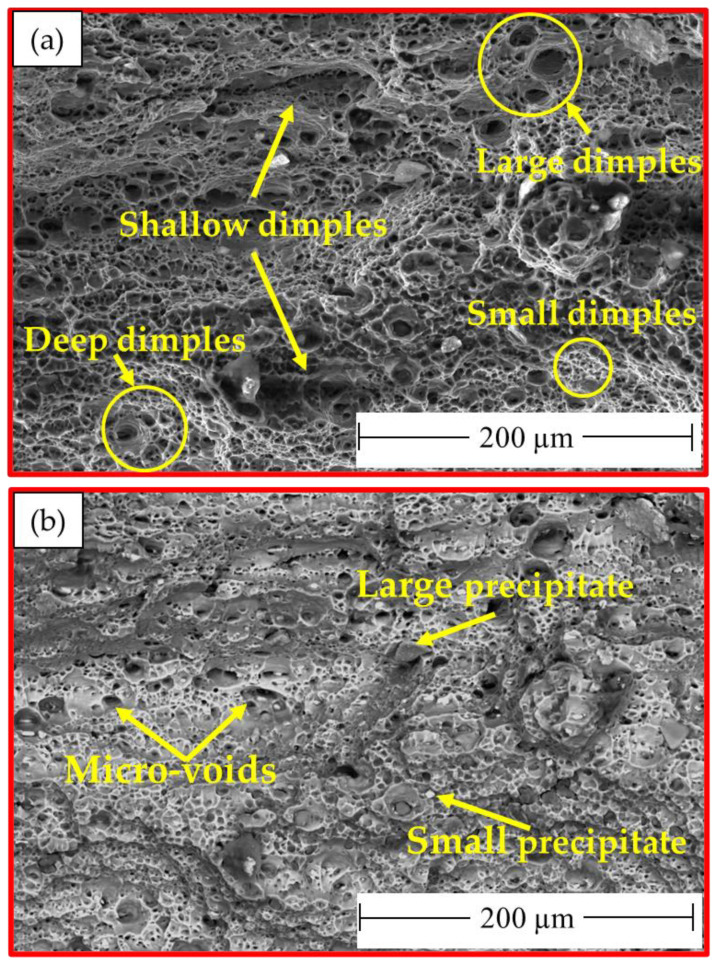
SEM fracture morphologies of AA5052-H32 BM: (**a**) ETD and (**b**) VCD.

The fracture morphologies of the AA 5052-H32 BM after tensile shear testing were investigated using two SEM detectors, namely ETD and VCD, as shown in Figure 11a,b, respectively. There were two fracture modes found (ductile and brittle). The ductile fracture mode was given by the AA5052 aluminum alloy in terms of various dimple shapes and sizes (Figure 11a), whereas the brittle mode was revealed by the existence of large precipitates of Mg_2_Si and Al_3_Fe [41]. In addition, microvoids were also observed due to the precipitate pull-out mechanism from the fracture surface (Figure 11b).

**Figure 12:**
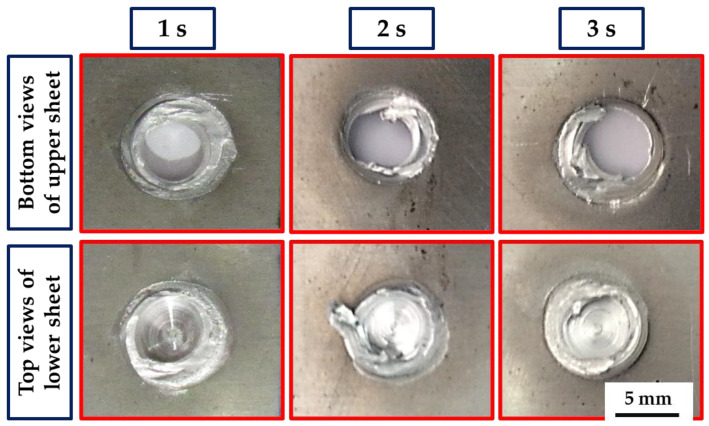
Photographs of the failed specimens of AA5052-H32 FSSW joints after tensile shear testing.

**Figure 13:**
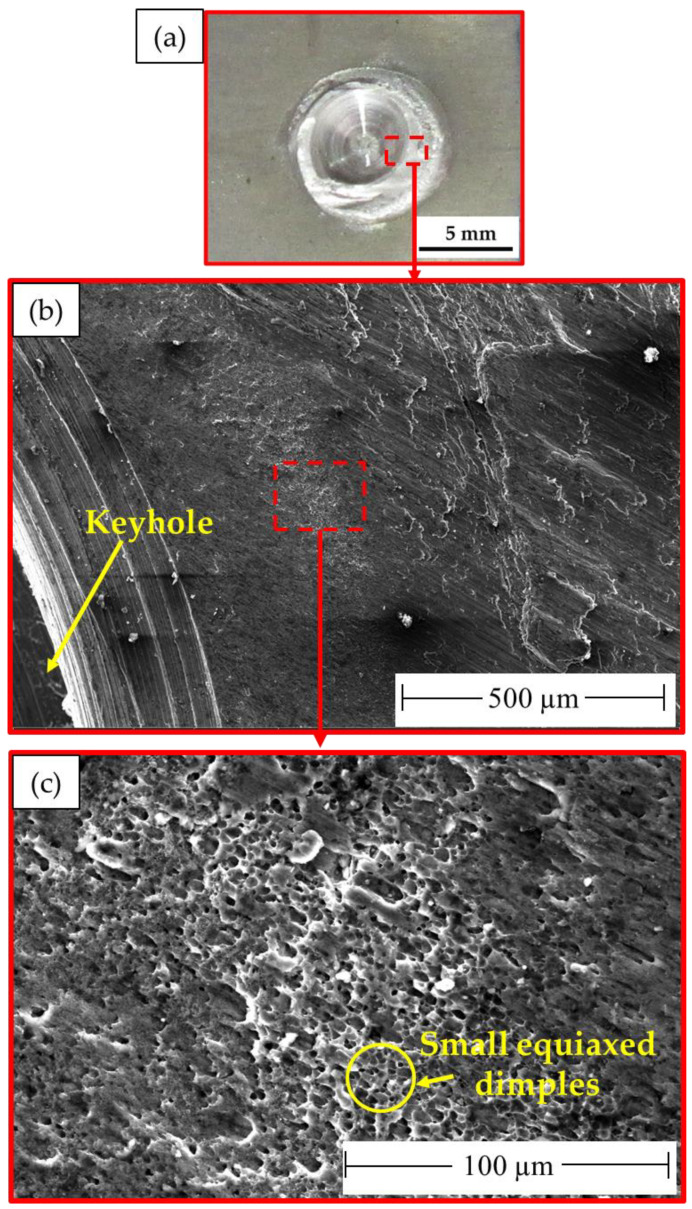
(**a**) Photograph of the top surface of the lower sheet of the joint FSSWed at 1 s and 500 rpm after tensile test and (**b**,**c**) different magnifications of SEM images showing the fracture surfaces of the failed joint. Note: dotted red lines enclosed area indicating where the micrographs were taken.

**Figure 14:**
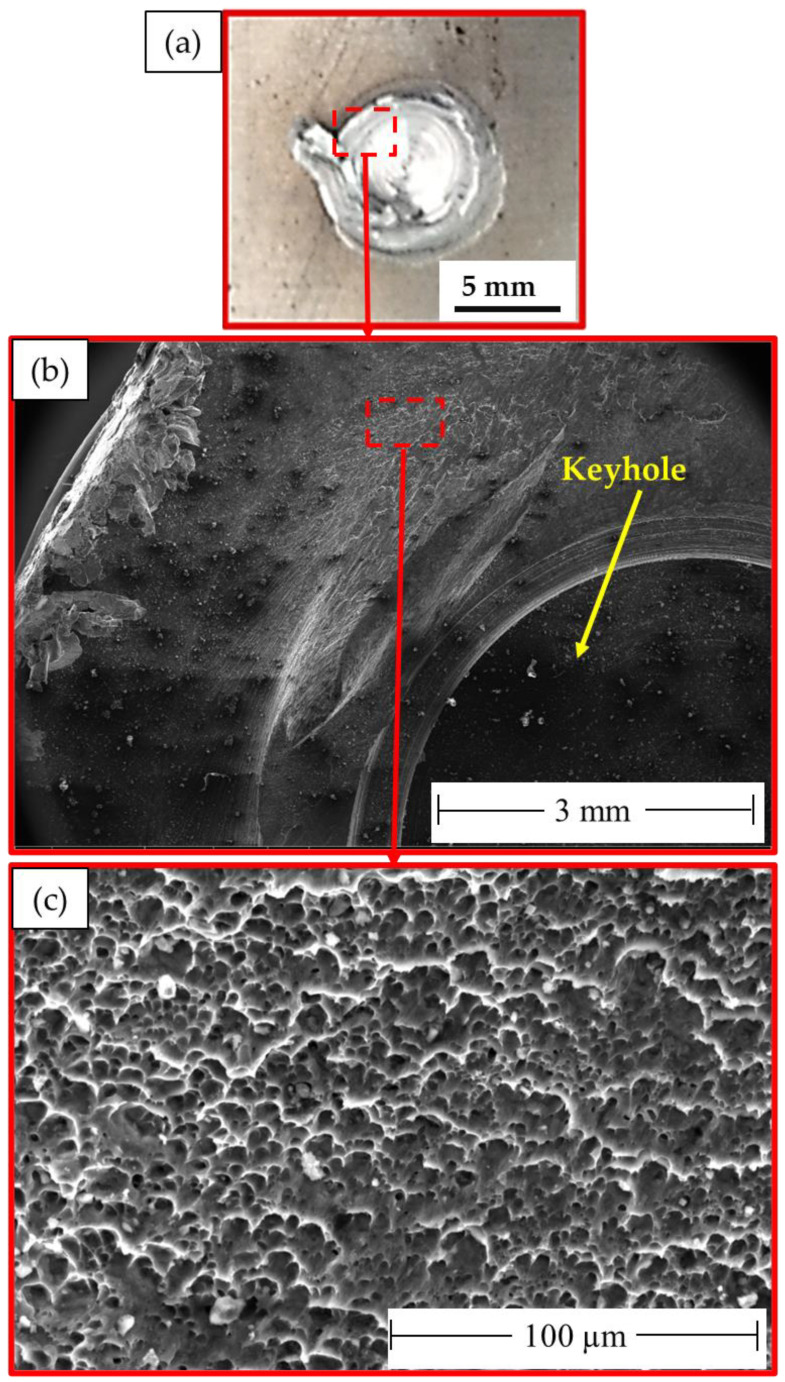
(**a**) Photograph of the top surface of the lower sheet of the joint FSSWed at 500 rpm and 2 s after tension test and (**b**,**c**) different magnifications of SEM fracture surfaces of the failed joint. Note: dotted red lines enclosed area indicating where the micrographs were taken.

**Figure 15:**
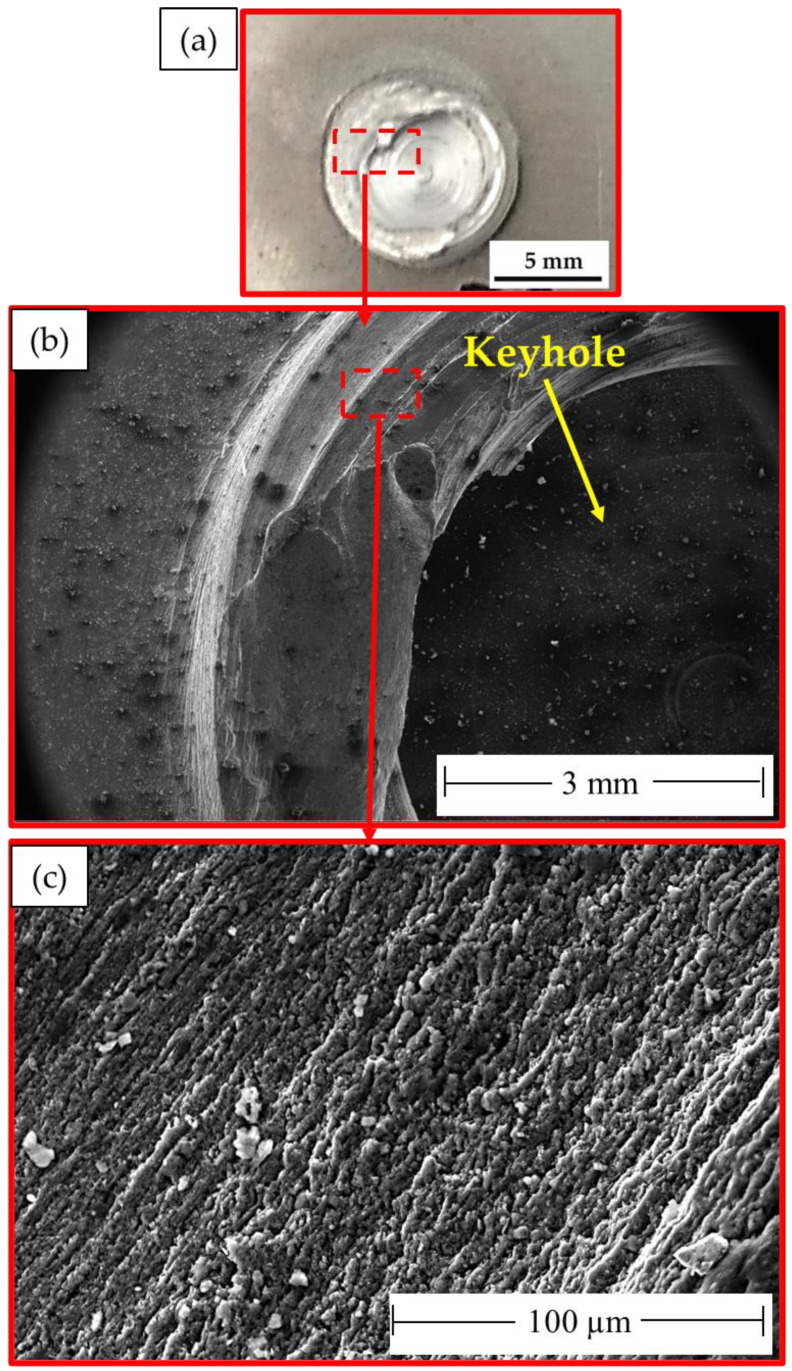
(**a**) Photograph of the top surface of the lower sheet of the joint FSSWed at 500 rpm and 3 s after tension test and (**b**,**c**) different magnifications of SEM fracture surfaces of the failed joint. Note: dotted red lines enclosed area indicating where the micrographs were taken.

It is important to note that the localization of the deformation inside the joint zones is typically responsible for the weld joint’s final fracture. The fracture surface of the failed stir zones was another aspect that might be used to identify the joint failure mode in FSSW. The produced spot-welded lap joints were separated during tensile shear testing. Photographs of the failed specimens of AA5052-H32 FSSWed joints after tensile shear testing are given in Figure 12. All the joints began to rupture at the edge of the SZ and develop along the depression in the AA5052-H32 upper sheet. Then, the joint was separated into two pieces, and the piece of upper sheet in the SZ was left in the piece of the lower sheet to yield a button shape. Figure 13, Figure 14 and Figure 15 show the SEM fracture morphologies of the lower sheets of the joints achieved at 500 rpm rotation rate and various dwell times of 1 s (Figure 13a–c), 2 s (Figure 14a–c), and 3 s (Figure 15a–c). The three FSSWed joints fractured mainly because of the shear fracture and tensile shear mechanisms based on the tensile shear loading test. The fracture morphologies of the three spot welds at the lower sheets displayed a ductile fracture mode (small equiaxed dimples), relative to the mixed modes of AA5052-H32 BM, indicating the grain refinement of the SZ during the FSSW process.

## 4. Grain Structure and Texture

**Figure 16:**
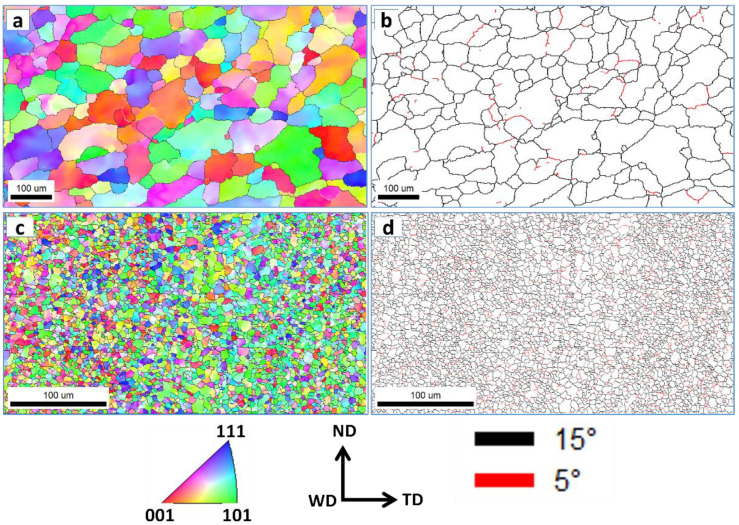
IPF maps and grain boundary maps of the AA5052-H32 BM (**a**,**b**) and the SZ after FSSW at 2 s and 500 rpm (**c**,**d**). The legend for IPF coloring is shown on the map, as well as the grain boundary legend.

**Figure 17:**
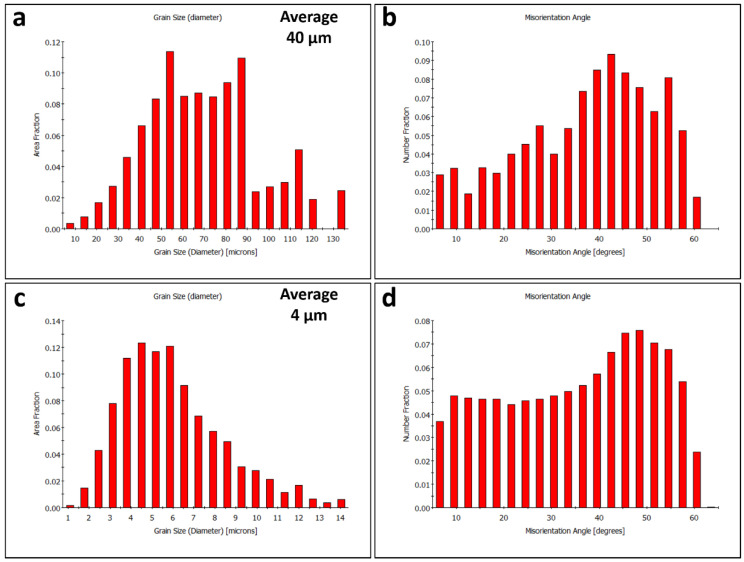
Histogram distributions of grain size and misorientation angle obtained from the OIM data given in Figure 18 for the AA5052-H32 BM (**a**,**b**) and the SZ after the FSSWed joint processed at 2 s and 500 rpm (**c**,**d**).

An EBSD investigation was carried out for both the AA5052-H32 BM and the SZ of the FSSWed specimen, which attained the highest tensile shear load-carrying capacity (processed at 2 s and 500 rpm) using a 2 µm and 0.5 µm step size, respectively, for almost the same grid area. The inverse-pole figure coloring (IPF) map and its corresponding grain boundary (GB) map of the AA5052-H32 BM are presented in Figure 16a,b. The grain structure was mainly composed of equiaxed coarse grains with a few small grains with a mean grain size of approximately 40 µm, as seen in the grain size distribution (Figure 17a). The grain boundary map was clearly dominated by high-angle grain boundaries (HAGB > 15°) with a limited number of low-angle grain boundaries (LAGB < 15°), as can be noted in Figure 16b. This is supported by the misorientation angle distribution depicted in Figure 17b. The IPF map and its corresponding GB map obtained inside the SZ of the FSSWed AA5052-H32 joint processed at 2 s and 500 rpm are presented in Figure 16c,d. It can be noticed that an equiaxed fine grain structure formed after FSSW with an average grain size of approximately 4 µm, as depicted in Figure 17c. A great reduction in average grain size can be observed from approximately 40 µm for the BM to about 4 µm after the FSSW process. This considerable grain size reduction was attributed to dynamic recrystallization during FSSW, which is considered a high-temperature severe plastic-deformation process [42,43,44]. In a study of AA5052 at different FSP parameters, Khraisheh et al. [45] showed a decrease in average grain size from 13.5 µm for the BM to approximately 4.5 to 1.7 µm based on FSP conditions. Additionally, Tiwan et al. [3] investigated the FSSW of AA5052-H112 using different FSSW parameters and reported significant grain size reduction without quantification of the grain size. The grain boundary map (Figure 16d) clearly shows a mixture of HABs and LABs with almost similar proportions. This indicates that the recrystallization process involves continuous dynamic recrystallization [42,46,47]. The misorientation angle distribution presented in Figure 17d also confirms the increase in the LAB fraction within the EBSD data.

**Figure 18:**
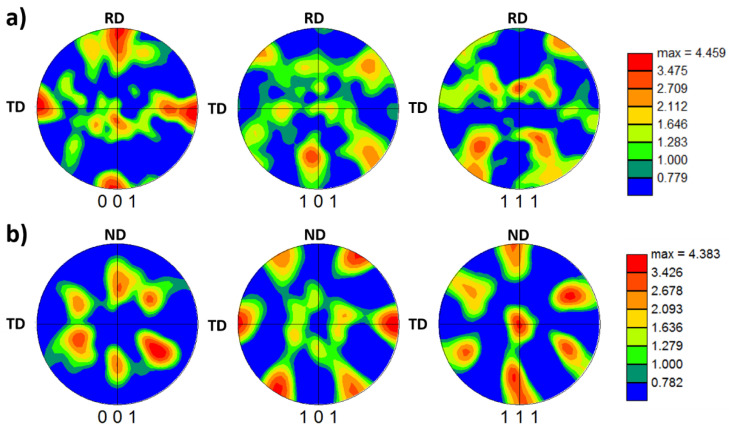
Pole figures (001, 101, and 111) obtained from the OIM data given in Figure 18 for the AA5052-H32 BM (**a**) and for the SZ after FSSW (**b**).

In terms of crystallographic texture, the 001, 101, and 111 pole figures are depicted in Figure 18 for the BM AA5052-H32 and the SZ after FSSW obtained from the maps illustrated in Figure 18. The texture of the BM (Figure 18a) is mainly dominated by the brass {110}<112> texture, which is one of the characteristic texture components for hot-rolled aluminum alloy. However, the texture in the SZ of the FSSWed material (Figure 18b) is dominated by a relatively strong simple shear texture with B/-B texture components at the ideal position after data rotation to align the shear reference axes with the welding reference axes [48].

## 5. Conclusions

Strips of 2 mm thick AA5052-H32 were FSSWed in this work at a rotation rate of 500 rpm and different dwell times of 1, 2, and 3 s. The produced joints were evaluated regarding the grain structure, hardness, maximum tensile shear load, and fracture morphologies. The main findings are as follows:

1. The used FSSW parameters of a fixed rotation rate of 500 rpm and different dwell times of 1, 2, and 3 s succeeded in joining 2 mm sheet thicknesses of 5052-H32 in spot lap joints.
2. All the FSSWed AA5052-H32 joints showed an enhancement in the hardness of the SZ and TMAZ relative to the BM. Moreover, the SZ gave higher hardness values than those given by the TMAZ and HAZ.
3. A reduction in BM grain size of 40 µm to 4 µm after FSSWed AA5052-H32 at 2 s and 500 rpm. This considerable grain size reduction is attributed to the grain refining that occurred during the FSSW process, which is mainly a high-temperature severe plastic-deformation process.
4. The FSSWed joint at 2 s and 500 rpm acquired the maximum SZ hardness value of 102.3 ± 3 HV0.5 and a maximum tensile shear load of 4330 ± 30 N among the manufactured joints.

## Figures and Tables

**Table 1:** Chemistry composition of the AA5052-H32 alloy material.

Elements	Mg	Fe	Mn	Si	Ti	Cu	Zn	Cr	V	Al
(wt.%)	2.490	0.258	0.091	0.127	0.017	0.001	0.200	0.194	0.001	Bal.

**Table 2:** Mechanical properties of the AA5052-H32 alloy starting material.

Alloy	Hardness (Hv)	Yield Tensile Strength (MPa)	Ultimate Tensile Strength (MPa)
AA5052-H32	69 ± 2	193 ± 3	229 ± 4

## Data Availability

Data are available upon request through the corresponding author.
